# Efficacy of avocado seed extract in preventing, inhibiting, and eliminating *Prevotella intermedia* biofilms: An *in vitro* study

**DOI:** 10.14202/vetworld.2025.408-418

**Published:** 2025-02-19

**Authors:** Nur Ariska Nugrahani, Maulita Misi Nurilyana, Imam Agus Faizal, Mahmud Kholifa, Ikmal Hafizi

**Affiliations:** 1 Department of Oral Biology, Faculty of Dentistry, Muhammadiyah University of Surakarta, 57141, Surakarta, Indonesia; 2 Department of Applied Bachelor's Degree of Medical Laboratory Technology, Faculty of Pharmacy, Science, and Technology, Al-Irsyad University of Cilacap, 53223, Cilacap, Indonesia; 3 Department of Orthodontics Dentistry, Faculty of Dentistry, Muhammadiyah University of Surakarta, 57141, Surakarta, Indonesia

**Keywords:** avocado seed extract, biofilm, natural agent, periodontitis, *Prevotella intermedia*

## Abstract

**Background and Aim:**

*Prevotella intermedia* is a significant contributor to periodontitis, capable of forming biofilms that resist antibiotics and complicate treatment. Avocado seeds (*Persea americana Mill*.) are rich in bioactive compounds, including flavonoids, tannins, saponins, and alkaloids, which exhibit potential antibiofilm activity. This study aims to evaluate the efficacy of avocado seed ethanol extract in preventing biofilm attachment, inhibiting biofilm formation, and eradicating established biofilms of *P. intermedia* *in vitro*.

**Materials and Methods:**

A post-test-only control group design was employed using *P. intermedia* (ATCC 25611). Ten groups were included: Bacterial and negative controls, a positive control (chlorhexidine), and experimental groups with ethanol extract concentrations (3.25%–9.25%). Biofilm activity was assessed using 96-well microtiter plates, crystal violet staining, and optical density measurements at 595 nm to determine the minimum biofilm prevention (MBPC), inhibition (MBIC), and eradication concentrations (MBEC). Statistical analysis was conducted using one-way ANOVA and Bonferroni *post hoc* tests.

**Results:**

Biofilm assays showed a dose-dependent increase in antibiofilm efficacy. The highest attachment prevention (82.67%), biofilm formation inhibition (84.26%), and biofilm eradication (86.04%) were observed at 9.25%. Significant differences (p < 0.05) were found between the extract and negative control groups, with no significant differences (p > 0.05) between the 8.25%–9.25% extracts and chlorhexidine. The MBPC50, MBIC50, and MBEC50 were identified at a concentration of 6.25%, achieving >50% efficacy in biofilm prevention, inhibition, and eradication.

**Conclusion:**

Avocado seed ethanol extract demonstrated significant antibiofilm properties against *P. intermedia*, comparable to chlorhexidine at higher concentrations. The bioactive compounds – flavonoids, tannins, saponins, and alkaloids – likely contributed to these effects through mechanisms such as quorum sensing inhibition, disruption of bacterial adhesion, and destabilization of biofilm structures. These findings highlight avocado seed extract as a promising natural alternative for managing periodontitis-related biofilm infections.

## INTRODUCTION

The primary pathogen causing periodontitis is *Prevotella intermedia*, a black-pigmented obligate anaerobic Gram-negative bacillus [1, 2]. *P. intermedia* is present in the subgingival plaque of patients with periodontitis and has a principal virulence component, lipopolysaccharide (LPS), which binds to host cells, including Toll-like receptors on immune cells, thereby eliciting inflammation. Inflammatory mediators include many pro-inflammatory cytokines, including interleukin (IL)-1, IL-6, and tumor necrosis factor-alpha, which aid in tissue repair; nevertheless, their overabundance may lead to tissue injury. Periodontal tissue comprises osteoclasts that resorb bone, derived from monocytes and macrophages that fuse in response to molecular signals, such as macrophage-colony stimulating factor and receptor activator of NFκB ligand [2]. Periodontitis may increase chronic inflammation, resulting in the growth and activation of osteoclasts, which degrade alveolar bone structure through osteoclastic activity. *P. intermedia* contains a LPS-virulence component that stimulates host cells to generate inflammatory mediators, facilitating osteoclast formation and activation, and leading to alveolar bone resorption [3]. This bacterium can also form biofilms, making it difficult to eliminate the affected area [1]. Biofilms comprise a combination of microorganisms that reside in self-generated exopolysaccharides [4]. Biofilm production involves cell attachment, intercellular adhesion and proliferation, growth, maturation, and dispersion. Quorum sensing, the mechanism by which bacteria and cells interact, is an integral aspect of this system, both interspecies and intraspecific, and starts at the stages of cell proliferation and development [5, 6].

*P. intermedia* serves as a secondary colonizer for biofilm formation, emergence, and proliferation following initial colonization by primary colonizers. *P. intermedia* attaches to primary colonizers through aggregators, facilitating the formation of secondary colonies [5, 7, 8]. This bacterium possesses an exopolysaccharide virulence factor that serves as the primary component in biofilm formation and functions to establish and safeguard biofilms against antibacterial agents and immune responses [5, 9]. These bacteria also secrete the extracellular DNA (eDNA) essential for biofilm formation [10]. Biofilms enable bacteria to withstand environmental stresses, including antibiotic resistance; thus, treating infections by biofilm-associated bacteria is challenging. Antibiotics for treating diseases associated with biofilms exhibit reduced efficacy and often require elevated dosages, posing potential health risks. This has created a demand for antibiofilm agents capable of addressing biofilm-related issues [11, 12]. Antibiotics can impede biofilm formation and either suppress or eradicate existing biofilms [12, 13]. Phytochemicals, specifically secondary metabolites derived from various plants, serve as natural antibiofilm agents that exhibit antibiofilm activity. The antibiofilm activity of phytochemical agents operates through several mechanisms, including interference with quorum sensing and cell signaling, inhibition of bacterial adhesin, suppression of genes associated with biofilm formation, reduction of exopolysaccharide production, and induction of dispersion by disrupting exopolysaccharide [14, 15]. Avocado seeds are natural substances with potential applications as antibiofilm agents. Avocado seeds are natural substances with the potential as antibiofilm agents due to their high concentrations of bioactive compounds and antimicrobial properties. The selection of avocado seeds was based on their historical application in traditional medicine and recent scientific research demonstrating their effectiveness against microbial pathogens. Avocado seeds possess secondary metabolite compounds that exhibit antibacterial and antibiofilm activities, including flavonoids, tannins, saponins, and alkaloids [14, 16]. Flavonoids interfere with microbial cell membranes and prevent biofilm formation. Tannins exhibit astringent properties that inhibit bacterial adhesion. Saponins induce cell lysis by enhancing cell membrane permeability. Alkaloids disrupt bacterial metabolism and diminish biofilm viability. Avocado seed contains compounds that are effective as natural alternatives to bacterial biofilms.

This study aimed to evaluate the effects of avocado seed (*Persea americana* Mill.). Ethanol extract prevents, inhibits, and destroys *P. intermedia* biofilms *in vitro*.

## MATERIALS AND METHODS

### Ethical approval

This study did not involve experiments on humans or animals, and ethics committee approval was not required.

### Study period and location

This study was performed from January to February 2024 at the UPT Materia Medica Batu Herbal Laboratory for the extraction and production of avocado seed ethanol extract and at the Research Center of Dental Medicine Laboratory, Faculty of Dentistry, Universitas Airlangga for biofilm formation and antibiofilm assessments.

### Study design

The design of study used a post-test-only control group and was entirely experimental. This study focused on undecayed avocado seeds, which are robust and free from fungi. The exclusion criterion was rotting or mold-infected avocado seeds. This study focused on *P. intermedia* (American Type Culture Collection [ATCC] 25611, USA). The study included ten groups: a bacterial control, a negative control (equivalent), a positive control (chlorhexidine), and seven experimental groups with ethanol extract concentrations of avocado seeds varying from 3.25% to 9.25%. Each group was replicated quintuple times.

### Preparation of avocado seed ethanol extract

Seventy percent ethanol was used to remove the avocado seeds during maceration. The maceration findings were transformed into a viscous extract by solvent separation through evaporation using a vacuum rotary evaporator at 50°C for 4 h. The concentrated extract was further diluted to concentrations of 3.25%, 4.25%, 5.25%, 6.25%, 7.25%, 8.25%, and 9.25%.

### Preparation of *P. intermedia* suspension

*P. intermedia* ATCC 25611 was inoculated into a Brain Heart Infusion Broth (BHIB) medium (Oxoid, UK Cat. No. CM1135) test tube, homogenized, and incubated for 24 h at 37°C. The turbidity of the bacterial suspension was equal to that of a standard solution of 0.5 Mc Farland.

### Biofilm formation test

The test and control wells of a 96-well flat-bottom microplate received 200 μL *P. intermedia* suspension, followed by supplementation with negative and positive controls. The solution was incubated at 37°C for 72 h without agitation. After incubation, the microplates were disposed of, and the cells were rinsed with phosphate-buffered saline (PBS). After 200 μL of 0.1% crystal violet was applied to each well, the biofilms were incubated at ambient temperature for 20 min. Crystal violet is a prevalent biofilm stain. It interacts with biofilm cells and extracellular polymeric substances (EPS) to enhance microscopic visibility. Absorbance measurements, which indirectly estimate biofilm biomass, were used to assess crystal violet adherence. Staining is essential for reproducible biofilm formation and density assessment. The tagged cells were removed from the microplates after three washes with PBS. However, they did not adhere to the microplate and were allowed to dry at ambient temperatures. The wells were then incubated with 200 μL of 96% ethanol at room temperature (22^o^C) for 20 min. Ethanol is used as both a disinfectant and a solvent. The wells were sterilized to eliminate microorganisms. The biofilms exhibited enhanced adherence to the wells when dehydrated and treated with ethanol. This method guarantees the accurate staining of biofilm presence and density. A microplate reader was used to evaluate the optical density (OD) of the test and control microorganisms at 595 nm wavelength. The strength of the test bacterial biofilm was assessed by comparing the OD of the isolate to the cutoff OD (ODcut = ODc – [3 times the standard deviation of ODc]). OD refers to the OD of the test microorganisms, and ODcut is the OD of the negative control. The results were classified as mild (ODcut < OD isolate ≤ 2×ODcut), moderate (2×ODcut < OD isolate ≤ 4×ODcut), strong (OD isolate > ODcut), or non-biofilm (OD isolate ≤ ODcut) [17].

### Biofilm adhesion prevention test

The test wells contained 200 μL of the avocado seed ethanol extract at concentrations of 3.25, 4.25, 5.25, 6.25, 7.25, 8.25, and 9.25%. In a 96-well flat-bottom microplate, 200 μL of equates was dispensed into the negative control wells, whereas 200 μL of chlorhexidine was introduced into the positive control wells. The wells were then incubated for 60 min at 37°C. The contents of the microplates were discarded, washed 3 times with PBS, and dried. *P. intermedia* solution (200 μL) was added to each well and incubated for 72 h at 37°C. The microplate contents were discarded again, and PBS was used to wash the plates 3 times. Subsequently, 200 μL of 1% crystal violet was added to each microplate well to stain the biofilm, which was then incubated at 22^o^C for 20 min. Following three PBS washes to eliminate tagged but unbound cells, the contents of the microplates were air-dried at ambient temperature. Each well was filled with 200 μL 96% ethanol and incubated at 22^o^C for 20 min. The OD used to determine the percentage of biofilm adhesion inhibition was measured by assessing biofilm formation at a wavelength of 595 nm using a microplate reader. The formula determines the percentage of inhibition of biofilm adhesion: (negative control OD-Test sample OD)/negative control OD) × 100%. The minimum biofilm prevention concentrations (MBPC), MBPC50, and MBPC90 were determined based on the percentage results. The results were obtained using minimal concentrations of extracts that inhibit biofilm adhesion by at least 50% and 90% [18].

### Biofilm formation inhibition test

One hundred microliters of ethanol extract (100 μL) from avocado seeds at concentrations of 3.25%, 4.25%, 5.25%, 6.25%, 7.25%, 8.25%, and 9.25% were added to the test wells, 100 μL of chlorhexidine was added to the positive control wells, and 100 μL of diluent was added to the negative control wells in a 96-well microplate. The test and control wells were administered 100-μL *P. intermedia* suspension, and 200 μL of the negative control and bacterial control wells were added. The wells were incubated for 72 h at 37°C. After disposal, the contents of the microplates were dried and washed 3 times with PBS. Subsequently, each well was filled with 200 μL 1% crystal violet and incubated at 22^o^C for 20 min. After three washes with PBS, the microplates were air-dried at ambient temperature. Each well was filled with 200 μL 96% ethanol and incubated at 22^o^C for 20 min. The OD and percentage inhibition of biofilm formation were assessed by measuring the biofilm produced at a wavelength of 595 nm using a microplate reader. The biofilm growth inhibition percentage was calculated as (negative control OD − test sample OD)/negative control OD × 100%. The minimum biofilm inhibitory concentrations (MBIC), MBIC50, and MBIC90 were determined based on the percentage results. The numbers were chosen based on the minimal concentrations of extracts that suppress biofilm development by at least 50% and 90%, respectively [19].

### Biofilm destruction test

A suspension of *P. intermedia* (200 μL) was introduced into the test wells, positive control, negative control, and bacterial control in 96-well microplates, followed by incubation for 72 h at 37°C. After incubation, the contents of the microplate were discarded, washed three times with PBS, and dried. Ethanol extracts (200 μL) from avocado seeds at concentrations of 3.25, 4.25, 5.25, 6.25, 7.25, 8.25, and 9.25% were added to the test wells. Subsequently, 200 μL of chlorhexidine was added to the positive control wells, and 200 μL of PBS was added to the negative control wells. The wells were incubated at 37°C for 60 min. The contents of the microplate were discarded again, followed by three washes with PBS. Subsequently, each well was filled with 200 μL 1% crystal violet and incubated at 22^o^C for 20 min. Following three PBS washes to eliminate tagged but unbound cells, the contents of the microplates were air-dried at ambient temperature. Each well was filled with 200 μL 96% ethanol and incubated at 22^o^C for 20 min. Biofilm generation was tracked using a microplate reader calibrated to 595 nm to determine the OD, which was used to calculate the percentage of biofilm clearance. The percentage of biofilm eradication was calculated ((negative control OD-test sample OD)/negative control OD × 100%). The minimum biofilm eradication concentration (MBEC), specifically MBEC50 and MBEC90, was determined using the lowest extract concentrations that could eradicate at least 50% and 90% of the biofilm, respectively, according to percentage results [20].

### Statistical analysis

The data obtained from the biofilm prevention, inhibition, and eradication tests were analyzed using IBM SPSS Statistics software version 27 (IBM Corp., NY, USA). The Shapiro–Wilk test was used to assess the normality of the data distribution, ensuring that the data followed a normal distribution, a prerequisite for parametric statistical analyses. p-value greater than 0.05 indicated that the data were normally distributed. Levene’s test was employed to evaluate the homogeneity of variances across the groups. Homogeneity is a critical assumption for conducting an analysis of variance (ANOVA), and p-value greater than 0.05 confirmed that the variances among the groups were equal.

A one-way ANOVA was performed to determine whether there were statistically significant differences among the treatment groups, including the bacterial control, negative control, positive control, and various concentrations of avocado seed ethanol extract. ANOVA was selected due to its ability to simultaneously compare the means of multiple groups. When the ANOVA indicated significant differences (p < 0.05), a Bonferroni *post hoc* test was conducted to identify specific group pairs with significant differences. The Bonferroni correction was applied to control for Type I errors due to multiple comparisons, ensuring robust and reliable conclusions.

Results are reported as mean ± standard deviation for optical density (OD) values across groups. Statistical significance was set at p < 0.05, and all tests were two-tailed. This comprehensive statistical analysis framework ensured the reliability of the findings, allowing for the accurate evaluation of the antibiofilm activity of avocado seed ethanol extract against *P. intermedia*.

## RESULTS

### Biofilm formation test

The isolate OD (0.6628) surpassed the ODcut value (0.56908) (Table 1); therefore, as ODcut < isolate OD ≤ 2 × ODcut, *P. intermedia* was categorized as a weak biofilm producer.

**Table 1:** *Prevotella intermedia* biofilm formation.

Treatment groups	Average ± Standard deviation
*Prevotella intermedia*	0.6628 ± 0.03228
Positive control	0.0858 ± 0.04020
Negative control	0.6586 ± 0.02984

### Biofilm adhesion prevention test

Table 2 presents the results of the OD measurements and the percentage of attachment inhibition in the biofilm adhesion prevention assay. The bacterial control group had the highest mean OD (0.7396. In the extract group, the maximum average OD was 3.25% (0.6532), whereas the minimum average OD was 9.25% (0.1272). The data indicate a trend toward decreasing OD with increasing extract concentrations (Table 2). The calculations showed that the maximum percentage of attachment prevention (82.67%) occurred at a concentration of 9.25%, with an average OD of 0.1272. The minimum percentage of attachment prevention (10.98%) was observed at a concentration of 3.25%, with an average OD of 0.6532 (Table 2). The MBPC was determined using these percentages. MBPC is a key measure in evaluating antibiofilm activity because it indicates the lowest concentration of a substance required to prevent the formation of biofilms by microorganisms [16]. MBPC50 was observed at a concentration of 6.25%, which yielded a value of 50.48%. Nonetheless, no MBPC90 was observed in the absence of the avocado seed ethanol extract, which exhibited a biofilm prevention rate of 90%.

**Table 2:** Optical density value of biofilm adhesion prevention test and percentage of *Prevotella intermedia* biofilm adhesion prevention.

Treatment groups	Sample replication	Average ± Standard deviation	% Prevention of biofilm adhesion
Control bacteria	5	0.7396 ± 0.03385	-
Negative control	5	0.7338 ± 0.03209	-
Positive control	5	0.2034 ± 0.05338	72.28
3.25%	5	0.6532 ± 0.02963	10.98
4.25%	5	0.5386 ± 0.03710	26.60
5.25%	5	0.4698 ± 0.02302	35.98
6.25%	5	0.3634 ± 0.06434	50.48
7.25%	5	0.3386 ± 0.03065	53.86
8.25%	5	0.2724 ± 0.02736	62.88
9.25%	5	0.1272 ± 0.02643	82.67

### Biofilm formation inhibition test

The OD dimensions of the microplate reader are listed in Table 3. The bacterial control group had the highest average OD of 0.7676. Within the extract group, 3.25% had the highest average OD (0.6440), whereas 9.25% had the lowest (0.1158). The average OD decreased from low to high extract concentrations (Table 3). The percentage inhibition of cell formation was determined based on the average OD. The results demonstrated that the ethanol extract of avocado seeds inhibited biofilm growth. In 9.25% of cells, the highest formation inhibition was 84.26% (OD 0.1158). In contrast, the lowest inhibition was 12.56% at 3.25% (OD 0.6434). The minimum biofilm-inhibitory concentration was determined based on these percentages. MBIC50 was observed at 6.25% with a rate of 50.67%; however, MBIC90 is not applicable as the ethanol extract of avocado seed inhibited biofilms at 90%.

**Table 3:** Optical density of biofilm formation inhibition test and percentage inhibition of *Prevotella intermedia* biofilm formation.

Treatment groups	Sample replication	Average ± Standard deviation	% Inhibition of biofilm formation
Control bacteria	5	0.7676 ± 0.03753	-
Negative control	5	0.7358 ± 0.03381	-
Positive control	5	0.1236 ± 0.05351	83.20
3.25%	5	0.6434 ± 0.02266	12.56
4.25%	5	0.5334 ± 0.03584	27.51
5.25%	5	0.4620 ± 0.01564	37.21
6.25%	5	0.3630 ± 0.06215	50.67
7.25%	5	0.3398 ± 0.03142	53.82
8.25%	5	0.2740 ± 0.03163	62.76
9.25%	5	0.1158 ± 0.02068	84.26

### Biofilm destruction test

The bacterial control group exhibited the highest average OD of 0.7938. Within the extract group, the 3.25% extract had the highest average OD of 0.6378, whereas the 9.25% extract had the lowest OD of 0.1072. The average OD of this group decreased for all extracts at various concentrations (Table 4). The percentage destruction technique used the average OD to evaluate the biofilm size that the avocado seed ethanol extract and positive control could eradicate. The positive control exhibited the highest destruction rate (87.56%). At 9.25%, the percentage was 86.04%, with an average OD of 0.1072, and 3.25%, with an average OD of 0.6378 (Table 4). The minimal biofilm eradication concentration (MBEC) was calculated using these percentages. The percentage of MBEC50 was 52.46%, and the concentration was 6.25%. No concentration of avocado seed ethanol extract eradicated biofilms up to 90%; therefore, no MBEC90 was present.

**Table 4:** Optical density of the biofilm destruction test and percentage of biofilm destruction in *Prevotella intermedia*.

Treatment groups	Sample replication	Average ± Standard deviation	% Biofilm destruction
Control bacteria	5	0.7938 ± 0.02083	-
Negative control	5	0.7682 ± 0.01973	-
Positive control	5	0.0956 ± 0.03500	87.56%
3.25%	5	0.6378 ± 0.01624	16.98%
4.25%	5	0.5444 ± 0.01629	29.13%
5.25%	5	0.4598 ± 0.01599	40.15%
6.25%	5	0.3652 ± 0.05658	52.46%
7.25%	5	0.3352 ± 0.03255	56.37%
8.25%	5	0.2740 ± 0.03163	64.33%
9.25%	5	0.1072 ± 0.01895	86.04%

### Statistical analysis

The results of the *post hoc* Bonferroni test are presented in Tables 5–8. The biofilm development test results demonstrated significant differences (p < 0.05) between the bacteria and the positive and negative control groups. The bacterial and harmful control groups did not differ significantly (p > 0.05) (Table 5). The results of the attachment prevention test (Table 6) indicated a significant difference (p < 0.05) between the 4.25% and 9.25% extract and negative control groups. The results of the creation and destruction inhibition tests (Tables 7 and 8) indicated a significant difference (p < 0.05) between the extract concentrations and the hostile control groups. The analysis indicated no significant difference between the 8.25% and 9.25% concentrations relative to the positive control in the prevention test.

**Table 5:** Results of the *post hoc* Bonferroni test on biofilm formation data.

Group	Bacteria	Negative control	Positive control
Bacteria	---	1.000	0.000*
Negative control	1.000	---	0.000*
Positive control	0.000*	0.000*	---

* (p < 0.05): Significant difference

**Table 6:** Results of the *post hoc* Bonferroni test on biofilm attachment prevention data.

	BC	CN	PC	3.25%	4.25%	5.25%	6.25%	7.25%	8.25%	9.25%
BC	---	1.000	0.000*	0.038*	0.000*	0.000*	0.000*	0.000*	0.000*	0.000*
CN	1.000	---	0.000*	0.076	0.000*	0.000*	0.000*	0.000*	0.000*	0.000*
PC	0.000*	0.000*	---	0.000*	0.000*	0.000*	0.000*	0.000*	0.285	0.127
3.25%	0.038*	0.076	0.000*	---	0.001*	0.000*	0.000*	0.000*	0.000*	0.000*
4.25%	0.000*	0.000*	0.000*	0.001*	---	0.291	0.000*	0.000*	0.000*	0.000*
5.25%	0.000*	0.000*	0.000*	0.000*	0.291	---	0.003*	0.000*	0.000*	0.000*
6.25%	0.000*	0.000*	0.000*	0.000*	0.000*	0.003*	---	1.000	0.022*	0.000*
7.25%	0.000*	0.000*	0.000*	0.000*	0.000*	0.000*	1.000	---	0.386	0.000*
8.25%	0.000*	0.000*	0.285	0.000*	0.000*	0.000*	0.022*	0.386	---	0.000*
9.25%	0.000*	0.000*	0.127	0.000*	0.000*	0.000*	0.000*	0.000*	0.000*	---

* (p-value <0.05): Significant difference. BC=Bacterial control, CN=Negative control, PC=Positive control

**Table 7:** *Post hoc* *Bonferroni test* data on biofilm formation inhibition.

	BC	CN	PC	3.25%	4.25%	5.25%	6.25%	7.25%	8.25%	9.25%
BC	---	1.000	0.000*	0.000*	0.000*	0.000*	0.000*	0.000*	0.000*	0.000*
CN	1.000	---	0.000*	0.0148	0.000*	0.000*	0.000*	0.000*	0.000*	0.000*
PC	0.000*	0.000*	---	0.000*	0.000*	0.000*	0.000*	0.000*	0.000*	1.000
3.25%	0.000*	0.014*	0.000*	---	0.001*	0.000*	0.000*	0.000*	0.000*	0.000*
4.25%	0.000*	0.000*	0.000*	0.001*	---	0.184	0.000*	0.000*	0.000*	0.000*
5.25%	0.000*	0.000*	0.000*	0.000*	0.184	---	0.006*	0.000*	0.000*	0.000*
6.25%	0.000*	0.000*	0.000*	0.000*	0.000*	0.006*	---	1.000	0.022*	0.000*
7.25%	0.000*	0.000*	0.000*	0.000*	0.000*	0.000*	1.000	---	0.346	0.000*
8.25%	0.000*	0.000*	0.000*	0.000*	0.000*	0.000*	0.022*	0.346	---	0.000*
9.25%	0.000*	0.000*	1.000	0.000*	0.000*	0.000*	0.000*	0.000*	0.000*	---

*(p < 0.05): Significant difference. BC=Bacterial control, CN=Negative control, PC=Positive control

**Table 8:** *Post hoc* Bonferroni *test results* for biofilm destruction data.

	BC	CN	PC	3.25%	4.25%	5.25%	6.25%	7.25%	8.25%	9.25%
BC	---	1.000	0.000*	0.000*	0.000*	0.000*	0.000*	0.000*	0.000*	0.000*
CN	1.000	---	0.000*	0.000*	0.000*	0.000*	0.000*	0.000*	0.000*	0.000*
PC	0.000*	0.000*	---	0.000*	0.000*	0.000*	0.000*	0.000*	0.000*	1.000
3.25%	0.000*	0.000*	0.000*	---	0.000*	0.000*	0.000*	0.000*	0.000*	0.000*
4.25%	0.000*	0.000*	0.000*	0.000*	---	0.002*	0.000*	0.000*	0.000*	0.000*
5.25%	0.000*	0.000*	0.000*	0.000*	0.002*	---	0.000*	0.000*	0.000*	0.000*
6.25%	0.000*	0.000*	0.000*	0.000*	0.000*	0.000*	---	1.000	0.001*	0.000*
7.25%	0.000*	0.000*	0.000*	0.000*	0.000*	0.000*	1.000	---	0.085	0.000*
8.25%	0.000*	0.000*	0.000*	0.000*	0.000*	0.000*	0.001*	0.085	---	0.000*
9.25%	0.000*	0.000*	1.000	0.000*	0.000*	0.000*	0.000*	0.000*	0.000*	---

*(p < 0.05): Significant difference. BC=Bacterial control, CN=Negative control, PC=Positive control

Furthermore, the study revealed no significant difference between the 9.25% concentration and the positive control in the formation and destruction tests, as supported by the supplementary *post hoc* Bonferroni test data from the three antibiofilm tests (p > 0.05). All tests demonstrated a statistically significant difference (p < 0.05) between the positive control group and the other concentration groups (Tables 6–8). The concentrations of 4.25%, 5.25%, 6.25%, 7.25%, and 8.25% showed no significant differences in the extract groups during the attachment prevention and suppression of biofilm formation tests, as determined by the *post hoc* Bonferroni test (p > 0.05). Tables 6 and 7 show the significant differences among the other groups (p < 0.05). Table 8 shows that the biofilm destruction test revealed no significant differences among the concentrations of 6.25%, 7.25%, and 7.25%, with a concentration of 8.25%, which also presented p-value exceeding 0.05. Tables 6–8 show significant differences (p < 0.05) among the groups.

## DISCUSSION

The biofilm formation test indicated an OD cutoff of 0.56908, whereas that of the isolate was 0.6628. The comparison of the two revealed that *P. intermedia* falls into the category of weak biofilm producers because it satisfied the condition ODcut < OD of isolation ≤ 2 × ODcut. Bacteria that formed biofilms could be categorized into four groups: those that did not develop biofilms (OD isolates ≤ ODcut), weak biofilm producers (ODcut < OD isolates ≤ 2 × ODcut), moderate biofilm producers (2 × ODcut < OD isolates ≤ 4 × ODcut), and strong biofilm producers (4 × ODcut < OD isolates) [17]. Five *P. intermedia* isolates were identified as poor biofilm producers based on the results of a biofilm formation test [21].

OD measurements were used to assess adhesion prevention, biofilm inhibition, and destruction. The OD indicates the quantity of biofilm formed. The three antibiofilm experiments showed that increased concentrations of avocado seed ethanol extract corresponded to higher percentages of biofilm prevention, inhibition, and destruction while simultaneously resulting in lower average OD values. The third *post hoc* Bonferroni test of the antibiofilm test (Table 6–8) revealed a negligible difference between the 6.25% and 7.25% extract groups (p > 0.05) and between the 7.25% and 8.25% extract groups (p > 0.05). The study findings demonstrated that the inhibition of biofilm development, prevention of adhesion, and destruction of biofilms increased from a concentration of 3.25% to an optimal concentration of 6.25% (p < 0.05). The figure indicates that increasing the concentration does not significantly influence the activities related to preventing attachment, inhibiting formation, and destroying biofilm, as the variation in OD values is minimal and statistically insignificant, suggesting a constant effect. A minimum inhibitory concentration of the avocado seed ethanol extract against *P. intermedia* of 3.125% and a minimum bactericidal concentration (MBC) of 6.25%. The MBPC, MBIC, and MBEC values in this study were determined using three antibiofilm tests corresponding to the MBPC, MBIC, and MBEC requirements, respectively [16]. Previous research by Mirzaei *et al*. [18] has indicated that MBPC50 and MBPC90 extracts exhibit the lowest concentrations capable of preventing biofilm adhesion by at least 50% and 90%, respectively. MBIC50 and MBIC90 represent the minimum concentrations of avocado seed extracts required to inhibit biofilm formation by at least 50% and 90%, respectively [19]. MBEC50 and MBEC90 represent the minimum concentrations of extracts required to eliminate at least 50% and 90% of an established biofilm, respectively [20].

The findings from the third antibiofilm test indicated that MBPC50, MBIC50, and MBEC50 were observed at a concentration of 6.25%. A concentration of 6.25% was identified as the minimum level of avocado seed ethanol extract that satisfied the criteria for MBPC50, MBIC50, and MBEC50, achieving attachment prevention, formation inhibition, and biofilm destruction rates of 50.48, 50.67, and 52.46%, respectively. No MBPC90, MBIC90, or MBEC90 values were observed due to the extract concentrations, as none satisfied the criteria for MBPC90, MBIC90, or MBEC90 (Tables 2–4). The ethanol extract of avocado seeds prevented, inhibited, and eradicated *P. intermedia* biofilms by >50%. The ethanol extract of avocado seeds prevented adhesion, inhibited formation, and destroyed *P. intermedia* biofilms at a concentration of 6.25%.

Concurrently, ethanol extracts from avocado seeds can prevent *Streptococcus mutans* and *Enterococcus faecalis* biofilms by 25% [22]. The ethanol extract of avocado seeds may prevent biofilm formation by *Pseudomonas aeruginosa* at a dosage of 8% [23]. The variation in extract concentrations capable of disrupting this biofilm arises from the classification of bacteria into groups with distinct cell wall compositions and structures, which influence their susceptibility to antibacterial agents. For instance, *S. mutans* is a Gram-positive bacterium, whereas *P. aeruginosa* and *P. intermedia* are Gram-negative bacteria [24,25]. Gram-positive bacteria possess a cell wall composed of a peptidoglycan layer that–20–80 nm in thickness [26]. The peptidoglycan content of these bacteria is 95%, resulting in a thick and rigid cell wall [27, 28]. In Gram-negative bacteria, the cell wall consists of a 7–8 nm thick peptidoglycan layer, comprising approximately 5%–10% of the total structure, resulting in a thinner cell wall than that in Gram-positive bacteria [26, 27]. Gram-negative bacteria possess porin, a water-filled protein channel in the outer membrane that allows entry of most antibacterial agents and hydrophilic metabolites [29, 30].

Bacterial biofilm formation contributes to resistance to antibacterial agents. Biofilms serve as the primary barrier against antibacterial agents, which may lead to bacterial resistance [31]. *S. mutans* and *P. aeruginosa* have a robust capacity for biofilm formation, whereas *P. intermedia* has a limited capacity for biofilm formation [21, 32, 33]. This indicates that *P. intermedia* has diminished resistance to antibacterial agents, requiring a much lower concentration of avocado seed ethanol extract to disrupt the biofilm of this bacterium. The *post hoc* Bonferroni results between the extract and positive control groups (Tables 6-8) further substantiated this. Chlorhexidine, a prevalent irrigation substance for curettage and the industry benchmark for antibiofilm agents in dentistry, has an efficacy equivalent to that of the 9.25% ethanol extract of avocado seeds as a positive control (p > 0.05) [34]. Nevertheless, the third antibiofilm assay demonstrated that 6.25% of the avocado seed ethanol extract inhibited, restrained, and eradicated at least 50% of the *P. intermedia* biofilms (Tables 2–4). This indicates that *P. intermedia* has little resistance to antibacterial agents, allowing for the disruption of its bacterial biofilm at concentrations far lower than those of the positive controls. Biofilm development involves bacterial adhesion to the host surface and other bacterial cells, which is facilitated by bacterial proteins and enzymes. *P. intermedia* contains an adhesin protein and glycosyltransferase enzyme that facilitate the adhesion of these bacteria. As a virulence factor, adhesin protein reduces the attachment of bacteria to the host surface and to other bacterial cells [35]. Furthermore, glycosyltransferases facilitate the transfer of sugar moieties, enabling bacterial adhesion to other bacterial cells and surfaces [36, 37]. During biofilm formation, additional virulence factors, including extracellular polymeric substances (EPS), are observed in *P. intermedia*. EPS is crucial for biofilm growth and formation [34]. EPS constructs biofilms, reinforces biofilm structures, and shields biofilms from human immune response and antibacterial agents [5, 9]. Moreover, eDNA is a critical structural component of the EPS matrix and is vital for biofilm growth. Microorganisms that generate biofilms actively release eDNA [10]. eDNA enhances EPS synthesis and biofilm formation, facilitates biofilm maturation, preserves EPS structural integrity, and bolsters resistance to antimicrobials and horizontal gene transfer [38–41].

Avocado seeds exhibit notable antibacterial, antibiofilm, and antifungal properties [16, 42]. The seeds of avocado exhibit no toxic properties, making them suitable for medicinal applications in the health sector [43]. This illustrates the antibacterial activity of the ethanol extract derived from avocado seeds against *P. intermedia*. The MBC of the avocado seed ethanol extract against *P. intermedia* was observed at a concentration of 3.125%, whereas its MBC was established at 6.25% [16]. Avocado seeds are regarded as potential antibiofilm agents due to their inherent properties. The findings of this study elucidate the efficacy of avocado seed ethanol extract against, impeding, and eradicating *P. intermedia* biofilms. Phytochemical examination has revealed significant findings [16]. The ethanol extracts of avocado seeds showed both qualitative and quantitative presence of secondary metabolites at varying concentrations: 0.48% flavonoids, 1.64% tannins, 2.59% saponins, and 2.59% alkaloids. Flavonoids, tannins, saponins, and alkaloids exhibit antibacterial and antibiofilm characteristics, which render them suitable natural antibiofilm agents [14, 16]. Each of these secondary metabolite functions as an antibiofilm agent through distinct mechanisms of action [14].

Flavonoids disrupt glycosyltransferase activity, reducing the production of glucans necessary for bacterial attachment. Consequently, glycosyltransferase cannot synthesize glucans, which serve as media for bacterial attachment. Flavonoids inhibit enzyme synthesis, which is responsible for producing autoinducer signaling molecules that modulate the quorum signaling process. This results in the suppression of quorum sensing, which hinders the development of microcolonies and the synthesis of extracellular polymeric substances, ultimately obstructing biofilm formation [44]. Flavonoids and tannins interact with bacterial adhesins to reduce their adhesion [45–47]. Tannins can impede biofilm formation due to their bacteriostatic properties, which damage bacterial membranes and hinder the synthesis of exopolysaccharide [48]. Saponin compounds can interact with eDNA, which is an integral element in biofilm formation, influencing its functionality. These changes reduce eDNA activity and its components, impeding biofilm formation [49]. Saponins effectively dismantle biofilms by disrupting the bonds between bacteria within the biofilm, thereby reverting them to their planktonic state. This transformation enhances the efficacy of existing antimicrobial compounds present [50]. Furthermore, saponins reduce extracellular polymeric substances within the biofilm matrix while simultaneously altering the integrity of the cell membrane, resulting in an unstable cell wall [51]. In the interim, alkaloids can impede quorum sensing [52, 53].

## CONCLUSION

This study demonstrated the significant antibiofilm activity of avocado seed ethanol extract against *P. intermedia*, a key pathogen in periodontitis. The extract showed dose-dependent efficacy in preventing attachment, inhibiting biofilm formation, and eradicating established biofilms. Notably, concentrations of 8.25% and 9.25% were comparable to chlorhexidine in efficacy (p > 0.05), achieving biofilm prevention, inhibition, and destruction rates of over 80%. The minimum concentrations for 50% efficacy (MBPC50, MBIC50, and MBEC50) were identified at 6.25%, indicating its potential as an effective natural alternative to synthetic antibiofilm agents.

The study’s strengths lie in its comprehensive evaluation of antibiofilm activities across multiple concentrations, rigorous statistical analysis ensuring reliable results, and the identification of bioactive compounds (flavonoids, tannins, saponins, and alkaloids) responsible for the observed effects. These findings contribute to the growing evidence supporting plant-based antibiofilm agents as safe and sustainable options for managing biofilm-associated infections.

However, the study has certain limitations. It was conducted *in vitro*, which may not fully replicate the complex conditions of the oral environment. The extract’s efficacy against other biofilm-forming oral pathogens was not assessed, and no evaluation of its potential toxicity or long-term effects was performed. In addition, the inability to achieve 90% biofilm prevention, inhibition, or eradication (MBPC90, MBIC90, and MBEC90) suggests the need for optimizing the extract’s formulation or combining it with other agents to enhance efficacy.

Future studies should focus on validating these findings *in vivo* to determine the extract’s clinical efficacy in managing periodontitis. Exploring its synergistic effects with other natural or synthetic antibiofilm agents and assessing its toxicity, pharmacokinetics, and long-term safety are critical steps toward its potential application as a therapeutic agent. Furthermore, investigating its activity against other biofilm-forming pathogens will broaden its scope and utility in clinical dentistry.

## AUTHORS’ CONTRIBUTIONS

NAN: Conceptualization, supervision, data curation, formal analysis, investigation, methodology, and writing-original draft, review, and editing. MMN: Data curation, formal analysis, investigation, methodology, and writing-original draft. IAF: Data curation, formal analysis, investigation, and methodology. MK: Formal analysis and review and editing. IH: Data curation, formal analysis, investigation, methodology, and review, and editing. All authors have read and approved the final manuscript.
